# Radiotherapy and the gut microbiome: facts and fiction

**DOI:** 10.1186/s13014-020-01735-9

**Published:** 2021-01-13

**Authors:** Jing Liu, Chao Liu, Jinbo Yue

**Affiliations:** Department of Radiation Oncology, Shandong Cancer Hospital and Institute, Shandong First Medical University and Shandong Academy of Medical Sciences, 440 Jiyan Road, Jinan, 250117 Shandong China

## Abstract

An ever-growing body of evidence has linked the gut microbiome with both the effectiveness and the toxicity of cancer therapies. Radiotherapy is an effective way to treat tumors, although large variations exist among patients in tumor radio-responsiveness and in the incidence and severity of radiotherapy-induced side effects. Relatively little is known about whether and how the microbiome regulates the response to radiotherapy. Gut microbiota may be an important player in modulating “hot” versus “cold” tumor microenvironment, ultimately affecting treatment efficacy. The interaction of the gut microbiome and radiotherapy is a bidirectional function, in that radiotherapy can disrupt the microbiome and those disruptions can influence the effectiveness of the anticancer treatments. Limited data have shown that interactions between the radiation and the microbiome can have positive effects on oncotherapy. On the other hand, exposure to ionizing radiation leads to changes in the gut microbiome that contribute to radiation enteropathy. The gut microbiome can influence radiation-induced gastrointestinal mucositis through two mechanisms including translocation and dysbiosis. We propose that the gut microbiome can be modified to maximize the response to treatment and minimize adverse effects through the use of personalized probiotics, prebiotics, or fecal microbial transplantation. 16S rRNA sequencing is the most commonly used approach to investigate distribution and diversity of gut microbiome between individuals though it only identifies bacteria level other than strain level. The functional gut microbiome can be studied using methods involving metagenomics, metatranscriptomics, metaproteomics, as well as metabolomics. Multiple ‘-omic’ approaches can be applied simultaneously to the same sample to obtain integrated results. That said, challenges and remaining unknowns in the future that persist at this time include the mechanisms by which the gut microbiome affects radiosensitivity, interactions between the gut microbiome and combination treatments, the role of the gut microbiome with regard to predictive and prognostic biomarkers, the need for multi “-omic” approach for in-depth exploration of functional changes and their effects on host-microbiome interactions, and interactions between gut microbiome, microbial metabolites and immune microenvironment.

## Background

Radiotherapy is a core modality used for the treatment of solid tumors [1]; more than 50% of patients with newly diagnosed cancer will receive radiotherapy over the course of the disease [2, 3], 60% with curative intent [4]. Although considerable progress has been made in the development of radiotherapy, its main limitations remain its effectiveness and safety. Clinical factors such as tumor size, disease stage, or tumor differentiation account for some of the heterogeneity in response to radiation among patients [5]. Accumulating evidence has also implicated biological factors in the ultimate outcomes of radiation therapy [6, 7], such as intrinsic radioresistance, hypoxia, inflammatory cell infiltration, and host immunity changes in the tumor microenvironment.

Radiotherapy is also associated with toxic side effects that negatively affect patients’ quality of life. Acute toxicities that may affect the patient’s ability to complete a treatment course include mucositis, dermatitis, cystitis, and bone marrow suppression. Chronic toxicities include fibrosis, vascular damage, or atrophy of the affected tissue or organ [4]. However, the incidence and severity of radiotherapy-induced toxicities vary substantially among patients [8]. Among the identified risk factors for developing toxicities are those related to therapy (radiation dose, volume, fraction, and site, and concomitant therapies) and those related to patients (age, sex, smoking, comorbid conditions, and genetic variations) [8, 9].

The gut microbiome can influence both the effectiveness of cancer treatment [10–13] and the severity of cancer treatment-induced gastrointestinal toxicities [14–18]. Microbiota niche can modify efficacy and toxicity profile of different onco-therapeutic treatment modalities from chemoradiotherapy to immunotherapy. Conversely, each of these treatment modalities has numerous effects on the gastrointestinal flora, causing changes in the gut microbial community that affects host morbidity and mortality [19]. The gut microbiome has been shown to affect the effectiveness and toxicity of various chemotherapies and immunotherapies through several mechanisms, primarily by modulating immune responses [20]. However, little is known about whether and how the gut microbiome modifies the response to radiotherapy [21]. Here we review “facts and fiction” regarding the nature of the interactions between radiotherapy and the gut microbiome. We discuss the potential influence of the gut microbiome on the antitumor effects of radiotherapy and its role in radiotherapy-induced gastrointestinal mucositis. We further explore the underlying mechanisms by which radiation and the gut microbiome participate in immunomodulation, and discuss potential treatments aimed at modifying the functions of the gut microbiome. We also summarized approaches to study the gut microbiome.

## Interplay between the gut microbiome and radiotherapy effectiveness

Gut microbiota may be an important player in modulating “hot” versus “cold” tumor microenvironment, ultimately affecting treatment efficacy [22, 23]. The gut microbiome is known to influence the effectiveness of various therapeutic strategies [24–27], including surgery, chemotherapy [27], androgen deprivation therapy [28] and immunotherapy [25, 29]. The role of the gut microbiome in radiosensitivity is a new concept that has generated substantial interest, but to date few original studies have had convincing results [21]. Relatively little is known about how the microbiome regulates the response to radiotherapy. What information is available is summarized in the following paragraphs.

### Bidirectional effects of radiation and gut microbiome composition

**Fig. 1:**
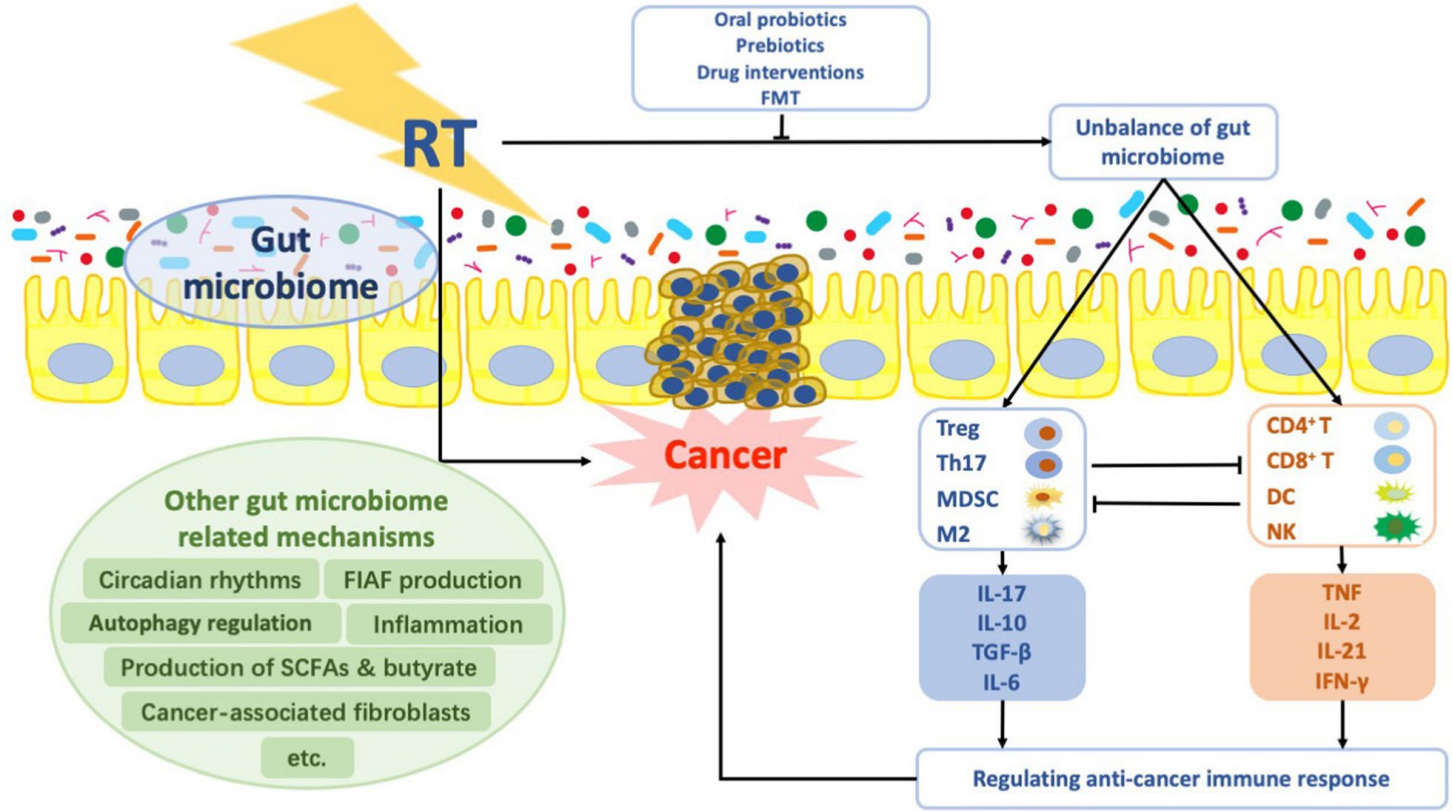
The potential mechanisms of the gut microbiome regulating the response to radiotherapy. *Notes*: Radiotherapy may reshape tumor microenvironment by microbiome, which involve the unbalance of anti-inflammatory and pro-inflammatory cell and their corresponding cytokines. Oral probiotics, prebiotics, drug interventions and FMT may maintain balance in the gut microbiome and then reshape the tumor microenvironment. Other gut microbiome related mechanisms on regulating the response to radiotherapy include circadian rhythms, FIAF production, autophagy regulation, inflammation, production of SCFAs and butyrate and cancer-associated fibroblasts etc. *RT* radiotherapy, *DC* dendritic cells, *IL* interleukin, *NK* natural killer cells, *TGF* tumor growth factor, *MDSC* myeloid-derived suppressor cells, *TNF* tumor necrosis factor, *IFN* interferon, *FMT* fecal microbial transplant, *FIAF* fasting-induced adipose factor, *SCFA* short-chain fatty acids

The interaction of the gut microbiome and cancer therapies, including radiation, is a bidirectional function, in that anticancer treatments can disrupt the microbiome (e.g., promoting dysbiosis) and those disruptions can influence the effectiveness of the anticancer treatments (Table 1). Kim et al., in characterizing the mouse gut microbiome, found that radiation causes significant changes in both the abundance and diversity of that microbiome, with increases in *Alistipes* and decreases in *Mucispirillum* genera [30]. A clinical study showed that pelvic radiotherapy resulted in remodeling of the overall gut microbiome composition, with a 10% decrease in *Firmicutes* and a 3% increase in *Fusobacterium* phyla [16]. A study [31] analyzing 45 fecal samples from patients with rectal cancer before concurrent chemoradation showed Bacteroidales (Bacteroidaceae, Rikenellaceae, Bacteroides) were relatively more abundant in patients with non-complete response (CR) than those with CR. *Duodenibacillus massiliensis* was linked with the improved CR rate. Generally, the most significant changes in the gut microbiome associated with cytotoxic chemotherapy or radiotherapy are increases in *Bacteroides* and *Enterobacteriaceae* and decreases in *Bifidobacterium*, *Faecalibacterium prausnitzii*, and *Clostridium* cluster XIVa [32]. Gut microbes can also shape normal and pathologic immune responses to cancer therapy. One group proposed that gut bacteria modulated the effects of chemotherapy via a host of mechanisms they called ‘TIMER’—that is, Translocation, Immunomodulation, Metabolism, Enzymatic degradation, and Reduced diversity [20]. A recent study [33] showed Higher alpha-diversity in the tumor microbiome of long-term survival patients and identified an intra-tumoral microbiome signature (*Pseudoxanthomonas-Streptomyces-Saccharopolyspora-Bacillus clausii*) highly predictive of long-term survivorship in both discovery and validation cohorts. Through human-into-mice fecal microbiota transplantation (FMT) experiments from short-term survival, long-term survival, or control donors, the tumor microbiome was differentially modulated, and tumor growth as well as tumor immune infiltration were affected. Logically, then, one could hypothesize that the gut microbiome also influences the immunostimulatory effects of radiotherapy (Fig. 1).

**Table 1:** Studies investigated interactions between the gut microbiome and radiotherapy effectiveness

Study	Study subjects	Treatment	Bacterial identification	Key findings
Nam et al. [16]	9 patients with gynecological cancer and 6 healthy controls	Pelvic RT 50.4 Gy/ 25 fractions/5 weeks	16 s RNA	The numbers of species-level taxa were severely reduced and the abundance of each community largely changed after RT
Kim et al. [30]	Male 8–10-week-old C57BL/6 mice	A single 8 Gy dose using a Cobalt 60 source irradiator	16 s RNA	Irradiation increased the level of the genera Alistipes in the large intestine and increased the level of the genus Corynebacterium in the small intestine
Jang et al. [31]	45 patients with rectal cancer	Pelvic RT, 50.0–54.0 Gy/ 25–30 fractions	16 s RNA	Differences in microbial community composition and functions were observed between CR and non-CR patients. Bacteroidales were relatively more abundant in patients with non-CR than those with CR
Uribe-Herranz et al. [34]	A melanoma model, a HPV E6/7-expressing lung and cervical cancer model in tumor-bearing mice	21 Gy using an XRAD320iX	16 s RNA	Gut microbiota can be modulated to improve RT-mediated antitumor responses. Vancomycin pretreatment enhanced the antitumor effects of RT in tumor-bearing mice
Cui et al. [36]	C57BL/6 mice	Total body irradiation exposure of 5 Gy	16 s RNA	Circadian rhythm is a key modulator in maintaining intestinal microflora balance. Mtnr1a and Mtnr1b might be involved in the circadian rhythm-shaped gut bacterial community
Crawford et al. [43]	CONV-R WT FVB/N mice	Mark I 137Cs irradiator (106 cGy/min for a total dose of 10–22 Gy)	Metabolomics	Fiaf deficiency results in loss of resistance of villus endothelial and lymphocyte populations to radiation-induced apoptosis

*CR* complete response, *RT* radiotherapy

Indeed, one group, seeking to explore whether the gut microbiota could modulate antitumor immune response after radiation to non-gut organs, used mouse models of B16-OVA melanoma and TC-1 lung/cervical cancer and found that the antibiotic vancomycin (which acts on gut bacteria) potentiated the radiation-induced antitumor immune response and inhibited tumor growth. This synergy depended on cross-presentation of tumor-specific antigens to cytolytic CD8 + T cells and on interferon-γ [34]. This group concluded that depletion of vancomycin-sensitive bacteria enhanced the antitumor activity of radiotherapy. Cui et al. [35] described a correlation between intestinal bacterial composition and radiosensitivity in an antibiotic-treated mouse model. The enteric bacterial composition of treated mice was significantly different from that of control mice, and the survival rate of the antibiotic-treated mice was significantly higher after irradiation.

### Potential mechanisms underlying gut microbiome disruptions, immune functions, and radiosensitivity

Evidence from both mouse models [36] and clinical studies [37] suggests an interaction between circadian rhythms, composition of the gut microbiome, and radiation sensitivity. Indeed, one literature review concluded that the time at which radiation was given can affect both local control and toxicity in patients with lung cancer [37].

Another hypothesis involves the link between radioresistance and autophagy regulation [38]. Digomann et al. [39] found that the expression level of some proteins involved in autophagy correlated with the clinical prognosis of patients with head and neck squamous cell carcinoma after chemoradiation [40]. The gut microbiome is also involved in autophagy regulation, and *Fusobacterium nucleatum* has been shown to have a role in chemoresistance to colorectal cancer by activating autophagy [41]. However, no studies have been published to date on the potential effects of gut microbiome composition on radiosensitivity via modulation of autophagy.

Inflammation may also have a role in the sensitivity or resistance of tumors to radiation. A component of the tumor microenvironment, cancer-associated fibroblasts, are involved not only in tumor initiation, progression, metastasis, and angiogenesis but also in immune modulation, including inflammation [42]; radiation increases the expression of TGF-β1, which activates cancer-associated fibroblasts. Other key regulators of the inflammatory process include CD8^+^ cytotoxic T cells and CD4^+^ T helper cells. Taken together, the complex inflammatory reactions launched by the immune system to an irradiated tumor and the surrounding stroma are neither wholly immuno-stimulatory nor immuno-suppressive.

Other insights about the microbial regulation of intestinal radiosensitivity come from studies of germ-free mice treated with whole-body gamma irradiation. One such study implicated fasting-induced adipose factor (FIAF), also known as angiopoietin-like 4 (ANGPTL4), a microbiota-regulated, epithelial-derived, secreted protein, in radioresistance, and suggested that FIAF may be useful as a gut radioprotector [43]. In another study, *Enterococcus faecalis*, *Clostridium perfringens*, *Bacteroides thetaiotaomicron*, and *Escherichia coli* were found to regulate FIAF production in colorectal cancer cell lines [44]. Transcription of ANGPTL4is regulated by peroxisome proliferator-activated receptors in response to bacteria that produce short-chain fatty acids [44, 45]. Indeed, probiotic bacteria shown to induce ANGPTL4 expression include *Streptococcus*, *Lactobacillus*, and *Bifidobacterium* spp, which led the authors to suggest that administering these probiotics may affect FIAF production and thus perhaps influence the course of colorectal cancer.

## Interplay between the gut microbiome and radiotherapy toxicity

Gastrointestinal mucositis is a particularly debilitating side effect of radiotherapy that can lead to significant declines in quality of life as well as treatment delays or dose reductions, which in turn can compromise treatment outcomes [32]. Radiotherapy-induced diarrhea is quite common, affecting more than 80% of cancer patients receiving pelvic radiotherapy [46]. However, some patients develop severe diarrhea after radiotherapy and some do not [15], suggesting that personalized treatment planning and identification of biomarkers with which to predict which patients are likely to respond to treatment or are at risk of developing severe toxicities would help to improve treatment outcomes.

The pathobiology of gastrointestinal mucositis has been described elsewhere [47, 48], but generally involves five stages [47]. Previous studies [49] have found that gut microbiota contributes to the pathogenesis of radiotherapy-induced gastrointestinal mucositis. Briefly, radiation initiates tissue injury followed by the upregulation and amplification of inflammation, which involves the production of proinflammatory cytokines. This leads to ulceration and enhanced inflammation due to interactions with microbial products crossing the breached epithelium. The final stage, healing, involves extracellular matrix signaling, proliferation of epithelial cells, and restoration of mucosal integrity.

Table 2 summarized studies investigated interactions between the gut microbiome and radiotherapy toxicity. Changes in the microbiome are important causative factors in the adverse effects of radiation enteropathy [18, 50]. Numerous studies have shown that radiotherapy causes major changes in the gut microbial composition [16–18, 51]. Several clinical studies of the microbiome before and after radiotherapy for gynecologic or lower gastrointestinal tract cancer all concluded that radiation induced significant changes in the microbiome profile [15–18, 52] including reducing the variation in the gastrointestinal and colonic microbiome. This reduced variation was notable among patients with gastrointestinal or gynecologic cancer who had diarrhea after irradiation compared with those who did not [16, 17]. Patients with radiation-induced diarrhea show greater changes in the gut microbiome community than patients who do not, and hence, the gut microbiome seems to be essential for protection against radiation-induced diarrhea [17, 53]. Patients who experience diarrhea were shown to have increased *Bacteroides*, *Dialister*, *Veillonella*, and unclassified bacterial species and reduced *Clostridium* XI and XVIII, *Faecalibacterium*, *Oscillibacter*, *Parabacteroides*, and *Prevotella* [15, 17]. Some evidence also suggests that patients undergoing radiotherapy have a high incidence of *Clostridium difficile* infection, which is associated with high mortality rates [54]. Research has revealed that gut microbiota composition can be used as a predictive marker for the development of radiotherapy-induced diarrhea and fatigue [49].

**Table 2:** Studies investigated interactions between the gut microbiome and radiotherapy toxicity

Study	Study subjects	Treatment	Bacterial identification	Key findings
Manichanh et al. [15]	10 patients undergoing pelvic RT	Pelvic RT 45–50 Gy/25 fractions/5 weeks	16 s RNA	Patients exhibiting diarrhea showed a progressive modification in microbial diversity
Nam et al. [16]	9 patients with gynecological cancer, data of 6 healthy controls	Pelvic RT 50.4 Gy/25 fractions/5 weeks	16 s RNA	Most patient suffered diarrhea symptom with dramatic change of gut microbial community after radiotherapy
Wang et al. [17]	20 patients undergoing pelvic cancer radiotherapy, 2 sequential stool samples were collected before and just after radiotherapy	Pelvic RT 50.4 Gy/ 25 fractions/5 weeks	16 s RNA	Microbial diversity, richness, and the *Firmicutes/Bacteroidetes* ratio were significantly altered prior to radiotherapy in patients who later developed diarrhea
Mitra et al. [18]	35 patients with locally advanced cervical cancer	Definitive radiation therapy, including external beam RT and brachytherapy	16 s RNA	Patients with high toxicity demonstrated different compositional changes during CRT in addition to compositional differences in Clostridia species
Gerassy-Vainberg et al. [51]	Female C57BL/6 J mice	4 fractions of 550 cGy with 24 h intervals	16 s RNA	Adherent microbiota from RP differed from those in uninvolved segments and was associated with tissue damage
Goudarzi et al. [52]	C57BL/6 J male mice	A whole-body dose of 0, 5 or 12 Gy X rays using an X-RAD 320 X-ray irradiator	16 s RNA and metabolomics	Statistically significant changes in the microbial-derived products such as pipecolic acid, glutaconic acid, urobilinogen and homogentisic acid
Riehl et al. [57]	FVB/N female mice and C57BL/6Jx129 COX-12/2, COX-22/2 mice	14 Gy total body at 0.96 cGy/min	Metabolomics	Lipopolysaccharide is radioprotective in the mouse intestine through a prostaglandin-dependent pathway
Riehl et al. [58]	C57BL/6 mice	12 Gy total body gamma irradiation	Metabolomics	TNFR1 and COX-2 expression to subepithelial fibroblasts plays an intermediate role in LPS-induced radioprotection in the intestine
Egan et al. [59]	Gene-targeted mice	8 Gy gamma irradiation	Metatranscriptomics	Selective preactivation of NF-kappa B through IKK in intestinal epithelial cells could provide a therapeutic modality that allows higher doses of radiation to be tolerated during cancer RT

*RT* radiotherapy

**Fig. 2:**
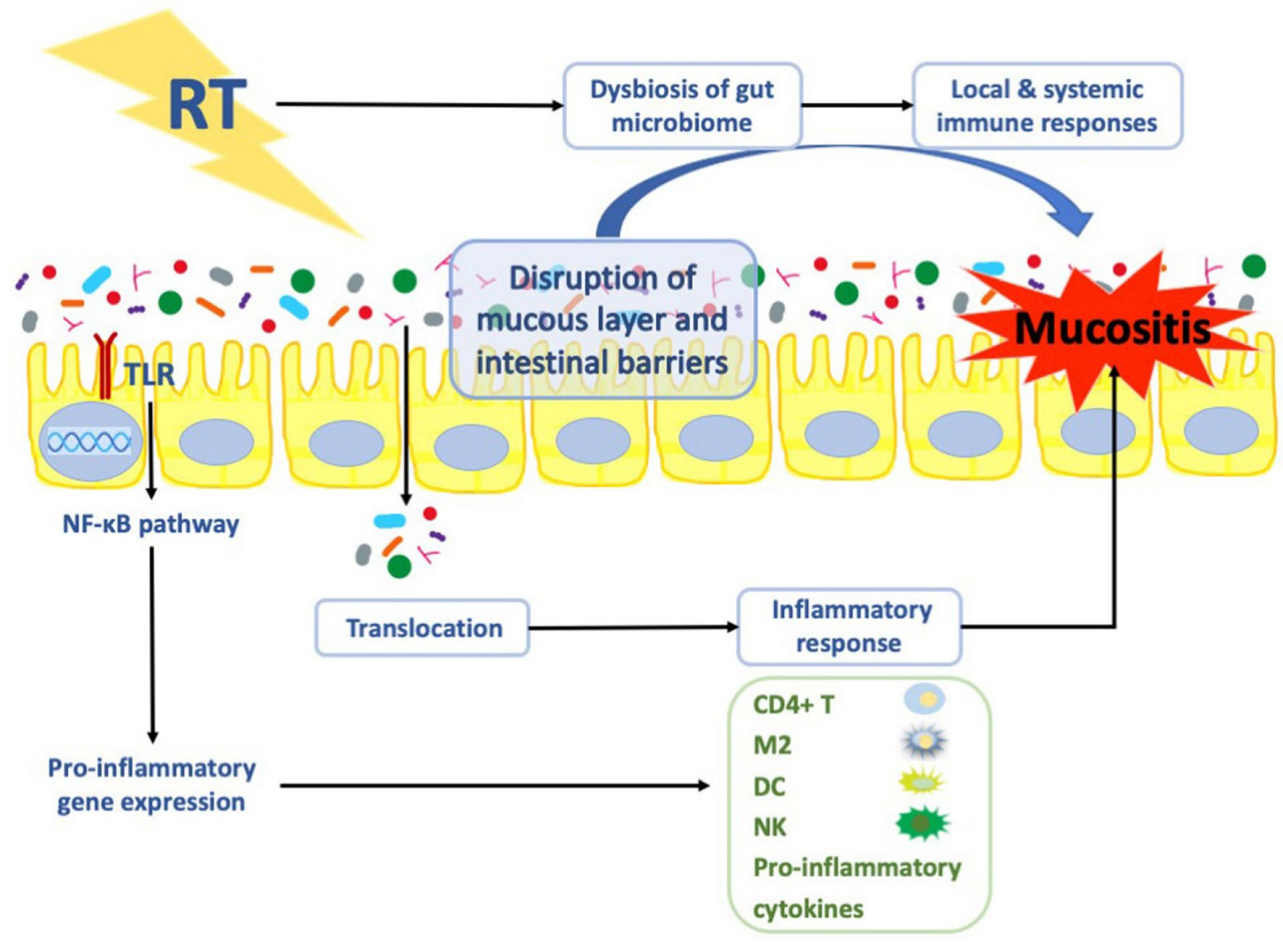
The potential mechanisms of the gut microbiome in radiation-induced intestinal mucositis. *Notes*: The gut microbiome can influence radiation-induced gastrointestinal mucositis mainly through two mechanisms: translocation and dysbiosis. Radiation disrupts the intestinal barriers and the mucus layer and causes bacterial translocation, resulting in activation of an inflammatory response. Dysbiosis, whether caused by radiation or other factors, can influence both local and systemic immune responses. Another potential mechanism by which TLR has protective effects against radiation is activation of NF-κB signaling, which is essential for the protection of the gut against radiation-induced apoptosis. *RT* radiotherapy, *TLR* toll-like receptor, *NF-κB* nuclear factor-kappa B, *DC* dendritic cells, *NK* natural killer cells

The influence of the gut microbiome on the pathogenesis of radiation-induced gastrointestinal mucositis [32] is mediated through modulation of the oxidative stress and inflammatory processes, intestinal permeability, mucus layer composition, epithelial repair and ability to resist harmful stimuli, and expression and release of immune effector molecules in the intestine [55]. The gut microbiome can influence radiation-induced gastrointestinal mucositis through two mechanisms (Fig. 2): translocation and dysbiosis. Radiation disrupts the intestinal barriers and the mucus layer and causes bacterial translocation, resulting in activation of an inflammatory response. Dysbiosis, whether caused by radiation or other factors, can influence both local and systemic immune responses.

The gut microbiome interacts with toll-like receptors (TLR) expressed on epithelial and immune cells to maintain intestinal homeostasis. Depletion of the gut microbiome in mice by using broad-spectrum antibiotics has been associated with increased susceptibility to methotrexate-induced gastrointestinal injury, which is suppressed by the administration of TLR2 ligands [14]. Conversely, knockout of TLR4 in mice has been shown to reduce irinotecan-associated pain and gut toxicity [56]. Also, the administration of lipopolysaccharide, a membrane component of Gram-negative bacteria, before radiation is known to protect intestinal crypts via induction of cyclooxygenase-2 and the production of prostaglandins [57]. Stimulation of TLR4-expressing cells by lipopolysaccharide also leads to the release of tumor necrosis factor (TNF) -α, which interacts with the TNF receptor on the surface of subepithelial fibroblasts, leading to the production of prostaglandins and reduction in radiation-induced apoptosis of epithelial stem cells [58]. Another potential mechanism by which TLR has protective effects against radiation is activation of nuclear factor-kappa B (NF-κB) signaling [55], which is essential for the protection of the gut against radiation-induced apoptosis. NF-κB activation also mediates the radioprotective effects of lipopolysaccharide [59], suggesting that TLRs may influence the intestinal response to radiation-induced epithelial damage through the NF-κB pathway.

### Potential therapies for gastrointestinal mucositis

Studies have begun to explore whether modifying the gut microbiome can maximize the response to treatment and minimize adverse effects [60]. Agents for modification studied to date include probiotics, prebiotics, or FMT, as described below.

#### Oral probiotics

Probiotics are defined as live microorganisms that have a beneficial role in cancer prevention and treatment by reducing the translocation of harmful bacteria, promoting intestinal immune barrier function and antipathogenic activity [61, 62]. Currently, *Lactobacillus*, *Bifidobacteria*, *Saccharomyces boulardii*, and *Bacillus coagulans* are the most common microbiome components used as probiotics [61, 62]. Synbiotics, representing a ‘bridge’ between prebiotics and probiotics, have been used to improve survival of probiotic bacteria during their passage through the upper intestinal tract [61].

A recent preclinical study of colorectal cancer cells [63] revealed that the combined application of *Propionibacterium freudenreichii* and TNF-related apoptosis-inducing ligand (TRAIL) increased proapoptotic gene expression and decreased antiapoptotic gene expression in those cells, suggesting that *P. freudenreichii* may be useful as an adjuvant for TRAIL-based colorectal cancer therapy. Probiotics have been shown to decrease the incidence and development of carcinogen-induced colorectal cancer in experimental models [64–66]. In a murine model of colorectal carcinoma, feeding the mice with engineered microbes and a diet of cruciferous vegetables led to significant tumor regression and reduced tumor occurrence [67]. Among patients undergoing surgery for colorectal cancer, oral probiotics have been shown to reduce tumor recurrence rates and to protect the physical and biological barrier functions of the intestinal mucosa [68, 69]. *Lactobacillus casei* has also been found to prevent atypia in colorectal tumors [70]. However, clinical reports indicated that use of probiotics or synbiotics had no measurable effect on gut barrier function, inflammatory response, or complications after surgery for colorectal cancer [71, 72]. Moreover, although synbiotic supplementation with *Bifidobacterium lactis* and resistant starch produced unique changes in the fecal microflora in another study of patients with colorectal cancer, it did not significantly alter any other fecal, serum, or epithelial biomarkers [73]. The authors of this study underscored the need to consider the patient’s family history and lifestyle, including diet, smoking, and other factors, before treatment with probiotics or synbiotics, and that further, in-depth research should be undertaken to gain a better understanding of the clinical value of these agents in colorectal cancer [74].

Indeed, studies of the effect of probiotics on radiation-induced gastrointestinal symptoms are difficult to evaluate, as they vary in the type of cancer patients recruited, the radiotherapy modalities used, the presence or absence of concomitant chemotherapy, end-point assessment, and the types of bacteria used as probiotics. A meta-analysis of six randomized controlled trials investigating probiotics and post-radiotherapy diarrhea suggested that oral probiotics could have beneficial effects in terms of reducing the incidence of diarrhea [75]. Although this is encouraging evidence, these clinical studies did not provide mechanistic details or objective evidence of the beneficial effect of probiotics on radiation-induced bowel injury. Moreover, further research is warranted with regard to how best to improve the formulation, administration, and absorption of probiotics or prebiotics-based therapies.

#### Prebiotics

In 2016, the International Scientific Association for Probiotics and Prebiotics updated the definition of a prebiotic as “a substrate that is selectively utilized by host microorganisms conferring a health benefit.” This definition expanded the concept of prebiotics to include non-carbohydrate substances and applications to body sites other than the gastrointestinal tract [76], thereby broadening the scope of prebiotics in research studies and clinical applications. As to their mechanism of action, both prebiotics and probiotics are thought to improve the integrity of the intestinal epithelial layer, and they may also increase resistance to pathogenic colonization. Probiotics, being new bacteria, are believed to enter the human intestinal tract and improve intestinal microecology, whereas prebiotics are intended to have a direct, regulated role in the gut microbiome.

Evidence from cell culture and animal models suggests that the consumption of prebiotics can inhibit colorectal carcinogenesis [77–79]. In healthy subjects, intervention trials indicated that consumption of palm, blackcurrant products, butylated starch, and wheat bran extract may have had a protective role in reducing the risk of developing colorectal cancer [80–83]. Another study showed that a diet rich in whole grains and dietary fiber was associated with a lower risk of *F. nucleatum*-positive colorectal cancer, but not *F. nucleatum*-negative colorectal cancer, suggesting that any association of diet with colorectal cancer risk significantly differed according to tissue *F. nucleatum* status [84]. In contrast, findings from a phase II chemoprevention trial did not provide convincing evidence that a 6-month intervention with prebiotic dietary fiber reduced the risk of developing colorectal cancer [85].

Notably, not all clinical studies of prebiotic or synbiotic therapies for colorectal cancer have shown conclusive results. Potential reasons for this include (a) differences in the pathogenesis of inflammation, genetic mutations, and epigenetic modifications in patients with colorectal cancer may result in prebiotics or synbiotics having multiple functions; (b) some specific species in the gut microbiome (pathogenic or not) could reduce or suppress the regulatory functions of prebiotics or synbiotics, or even “hijack” these agents to facilitate colorectal cancer progression under certain conditions; and (c) although prebiotics and synbiotics have important roles in modulating immune development and function and in maintaining balance in the gut microbiome, some cases of severe gut microbiome dysbiosis may not be controllable with prebiotics. Addressing these and other potential explanations may allow the use of oral prebiotics or synbiotics to prevent or control colorectal cancer in the future.

#### Drug interventions

Antibiotics are well known to affect the composition of the gut microbiome, but how these effects interact with the development and progression of colorectal cancer is less clear. In one study of heme-induced carcinogenesis in rats, antibiotics were found to suppress the microbiome by reducing crypt height and proliferation, thereby implicating the microbiome in heme-induced promotion of colorectal cancer [86]. Antibiotics such as anisomycin, prodigiosin, and salinomycin seem to inhibit the growth of colorectal carcinoma cells by targeting different molecular mechanisms [87–89]. Another study showed that treating mice bearing colon cancer xenografts with the antibiotic metronidazole reduced the *Fusobacterium* load, cancer cell proliferation, and overall tumor growth, which collectively suggested that antimicrobial interventions may be useful for patients with *Fusobacterium*-associated colorectal cancer [90]. Whether the anti-colorectal cancer properties of these drugs, present in natural microorganisms, are related to the function and balance of the gut microbiome is unclear, but they suggest an avenue for exploring and developing novel antibiotics or antibiotic peptides that are based on the human gut microbiome itself.

Other agents, including celecoxib, berberine, isoliquiritigenin, and curcumin, have also been found to decrease the incidence of colorectal tumorigenesis by modulating the gut microbiome [91–94]. Recent studies of the “fingerprint” of the human gastrointestinal tract microbiome involving the study of many complex bacterial ecosystems could push the development of narrow-spectrum antibiotics for use in treating colorectal cancer, as well as facilitating systems pharmacology and personalized therapeutics [95, 96].

#### FMT

Another potential means of manipulating the gut microbiota has been the use of FMT, in which a fecal suspension is transferred from healthy donors into the gastrointestinal tract of other individuals, with goal of curing specific conditions or diseases by reconstructing the normal function and immune system of the gut microbiome. FMT transplants can consist of fresh stools or frozen fecal capsules, or extracts of bacterial flora from normal fecal flora. Although direct evidence is lacking at present to support the use of FMT for treating colorectal cancer, fecal microbiomes isolated from patients with colorectal cancer have been shown to promote intestinal carcinogenesis in germ-free mice and in mice given a carcinogen [97, 98]. This indirect evidence suggests that FMT may be effective for preventing and treating colorectal cancer by its ability to improve the balance and function of the human gut microbiome. A clinical study [99] demonstrated that FMT might be safe and effective to improve intestinal symptoms and mucosal injury in patients with chronic radiation enteritis. Additionally, FMT is also shown to be an efficacious remedy to mitigate acute radiation syndrome. Recent study [100] confirmed that indole 3-propionic acid is a key intestinal microbiota metabolite corroborating the therapeutic effects of FMT to radiation toxicity.

Notably, the effects of FMT on the recipient immune system are complex and unpredictable, and the risk that FMT may lead to dissemination of unknown pathogens cannot be eliminated [101]. Numerous questions remain regarding the role of FMT, including the need to identify what makes a “good” donor, the optimal routes of administration, preparation of transplant materials, regulatory frameworks, and long-term effects [102, 103]. If we can identify favorable fecal microbiome composition, or safe and functionally well-defined bacterial strains, and use prebiotics as the “packaging material” for delivery, FMT may be an effective, low-burden supplement or alternative to chemoradiation in the near future.

Other novel approaches could include bioengineering the gut microbiome [104–106], the synthesis and delivery of genetically engineered probiotics [107, 108] or bacteriocins [105, 108, 109] or bacteriophages [110, 111] to modify the gut microbiome. Promisingly, the delivery of encoded nanobody antagonist of CD47 by tumor-colonizing bacteria increases activation of tumor infiltrating T cells, stimulates rapid tumor regression, prevents metastasis, and leads to long-term survival in a syngeneic tumor model. An abscopal effect was also induced by an engineered bacterial immunotherapy [106]. Additional research imperative to evaluate the potential of these engineered products for clinical application in the context of colorectal cancer.

## Approaches to studying the gut microbiome

The majority of published studies used 16S rRNA sequencing to investigate and compare the taxonomic distribution and diversity of gut microbiome between individuals. However, 16S rRNA only identify bacteria level other than strain level. The functional gut microbiome can be studied using methods involving metagenomics, metatranscriptomics, metaproteomics, as well as metabolomics. The above methods may provide significant functional information for network analyses, and identification of proteins and metabolites produced by gut microbiome.

Sequencing the collection of genomes present in an ecosystem is known as metagenomics. Shotgun metagenomics provides an enormous amount of valuable functional information down to the strain level and for all types of microorganisms, therefore is now widely applied [112, 113]. However, it is quite clear that the presence of a specific gene does not inform us about its gene expression patterns. Metatranscriptomics and metaproteomics can measure transcripts and proteins directly, and are becoming important approaches additional to metagenomics. Their combination enables identification of up and down-regulated genes under specific conditions. Nucleic acid sequencing is also applied in metatranscriptomics as metagenomics. Metaproteomics measures expressed proteins using high-resolution mass spectrometry [114]. Considering not all transcripts are ultimately translated into proteins, metaproteomics provides superior insight into gut microbial functionality as compared with metatranscriptomics. What’s more, metabolomics directly measures the metabolites produced by gut microbiome using analytical techniques including nuclear magnetic resonance spectroscopy or mass spectrometry. Profiling metabolomes of microbial metabolites during radiation therapy can provide valuable information on bi-directional radiation-microbiome interactions that may contribute to the identify the underlying mechanism of the communication between microbiome and host during radiation therapy.

Considering each ‘-omic’ technology provides its own unique perspective of the microbiome and its communication with the host, multiple ‘-omic’ approaches can be applied simultaneously to the same sample to obtain integrated results [115].

## Challenges and remaining unknowns for future research

The inter-individual variations seen in the response to radiation and in the severity of radiation-related toxic effects remain major challenges in the use of radiotherapy for cancer treatment. Considerable research effort has been devoted to identifying factors that could explain this variation, with particular interest expressed recently in how the gut microbiome influences radiation response and toxic effects. However, many unknowns still remain in attempts to clarify the complex, bidirectional relationship between the gut microbiome and radiation effects.

First, the role of the gut microbiome in radiosensitivity is a new concept that has generated a lot of interest, but few original studies have yielded convincing results. The mechanisms underlying how the gut microbiome influences radiosensitivity are still obscure, and much more research is needed to clarify the links between the gut microbiome and variations in radiotherapy response.

Second, radiotherapy is increasingly being combined molecular targeted therapy or immunotherapy in the treatment of solid tumors. The mechanisms underlying the synergistic effects of such combinations are a “hot topic” in research, and further information on how the gut microbiome participates in these effects is urgently needed to enhance radiation-based combined treatments for cancer. Because patients participating in clinical trials are already closely monitored, it will be important to include comprehensive microbiome assessments in this monitoring to fully understand the baseline microbiome in cancer patients and to study the effects of various therapies on specific bacterial families and their contribution to therapeutic outcomes.

Third, aspects of the microbiome could be used to predict cancer risk, recurrence, response to therapies, and survival—in other words, aspects of the gut microbiome could be useful as predictive and prognostic biomarkers. Future research to investigate the influence of the gut microbiome on the incidence and severity of radiotherapy-induced mucositis is warranted, with a view toward modulating the microbiome composition to improve cancer therapy outcomes.

Fourth, findings from most analyses of the gut microbiome undertaken to date have relied on next-generation sequencing. However, the presence of a gene or its transcript does not necessarily indicate protein expression; therefore, direct measurements of expressed proteins via meta-proteomics will be useful for providing precise functional information on the microbiome. Indeed, thorough examinations of the gut microbiome should include metaproteomic analysis, which can reveal both human and microbial functional changes indicative of the host-microbiome interactions. More recently, “microscomics” approach was conducted in human stool samples by transmission electron microscopy, which may further decline the inconsistencies observed with metagenomics and culturomics [116]. This is an exciting avenue for novel therapies.

**Fig. 3:**
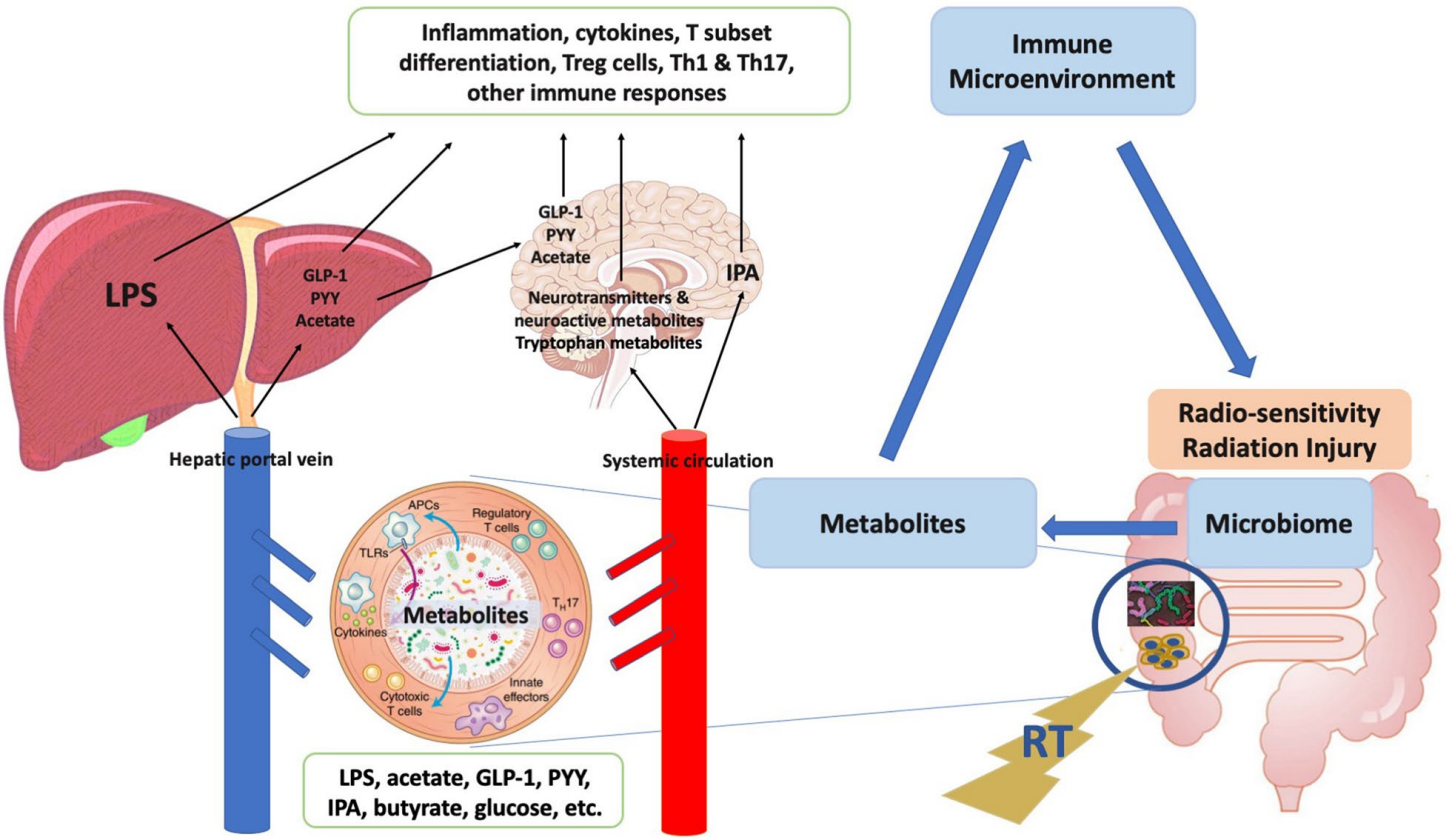
Crosstalks between gut microbiome, microbial metabolites and immune microenvironment may explain the underlying mechanism of gut immune alliance. *Notes*: Microbial metabolites induced by radiotherapy could enter the bloodstream transported to the liver, brain and other organs of the body. Immune microenvironment is thus changed and may modulate radio-sensitivity and radiation injury. *GLP-1* glucagon-like peptide-1, *PYY* peptide tyrosine tyrosine, *LPS* lipopolysaccharide, *IPA* indolepropionic acid, *APC* antigen-presenting cell, *TLR* toll-like receptor

Finally, previous studies showed that microbial metabolites produced locally can enter the bloodstream and act systemically (Fig. 3) [117, 118]. Crosstalk between gut microbiome, microbial metabolites and immune microenvironment may modulate radiosensitivity, which converting immunologically “cold” tumors to “hot”, and even “hot” tumors to “hotter”, ultimately affecting treatment efficacy. Immune microenvironment is also closely related to radiation injury. Radiation-induced toxicity may be predicted by potential metabolic biomarkers, and be reduced by oral nutritional approaches including changes in diet, probiotics, prebiotics, etc. Harnessing the interactions between gut microbiome, microbial metabolites and immune microenvironment is the current and future research directions of our research group, with more research outcome is to be expected.

## Data Availability

Not applicable.

## Abbreviations

TLR: Toll-like receptor
NF-κB: Nuclear factor-kappa B
Tregs: Regulatory T cells
TGF-β: Transforming growth factor-β
FIAF: Fasting-induced adipose factor
ANGPTL4: Angiopoietin-like 4
TNF: Tumor necrosis factor
TRAIL: Tumor necrosis factor-related apoptosis-inducing ligand
FMT: Fecal microbial transplantation
CR: Complete response

## References

[1] Ahmad SS, Duke S, Jena R, Williams MV, Burnet NG (2012). Advances in radiotherapy. BMJ.

[2] Jaffray DA (2012). Image-guided radiotherapy: from current concept to future perspectives. Nat Rev Clin Oncol.

[3] Delaney G, Jacob S, Featherstone C, Barton M (2005). The role of radiotherapy in cancer treatment: estimating optimal utilization from a review of evidence-based clinical guidelines. Cancer.

[4] Barnett GC, West CM, Dunning AM, Elliott RM, Coles CE, Pharoah PDP (2009). Normal tissue reactions to radiotherapy: towards tailoring treatment dose by genotype. Nat Rev Cancer.

[5] Begg AC, Stewart FA, Vens C (2011). Strategies to improve radiotherapy with targeted drugs. Nat Rev Cancer.

[6] Park SY, Lee CJ, Choi JH, Kim JH, Kim JW, Kim JY (2019). The JAK2/STAT3/CCND2 axis promotes colorectal cancer stem cell persistence and radioresistance. J Exp Clin Cancer Res.

[7] Buckley AM, Lynam-Lennon N, O'Neill H, O'Sullivan J (2020). Targeting hallmarks of cancer to enhance radiosensitivity in gastrointestinal cancers. Nat Rev Gastroenterol Hepatol.

[8] Bentzen SM, Overgaard J (1994). Patient-to-patient variability in the expression of radiation-induced normal tissue injury. Semin Radiat Oncol.

[9] Andreassen CN, Alsner J (2009). Genetic variants and normal tissue toxicity after radiotherapy: a systematic review. Radiother Oncol.

[10] Viaud S, Saccheri F, Mignot G, Yamazaki T, Daillère R, Hannani D (2013). The intestinal microbiota modulates the anticancer immune effects of cyclophosphamide. Science.

[11] Iida N, Dzutsev A, Stewart CA, Smith L, Bouladoux N, Weingarten RA (2013). Commensal bacteria control cancer response to therapy by modulating the tumor microenvironment. Science.

[12] Vétizou M, Pitt JM, Daillère R, Lepage P, Waldschmitt N, Flament C (2015). Anticancer immunotherapy by CTLA-4 blockade relies on the gut microbiota. Science.

[13] Sivan A, Corrales L, Hubert N, Williams JB, Aquino-Michaels K, Earley ZM (2015). Commensal Bifidobacterium promotes antitumor immunity and facilitates anti-PD-L1 efficacy. Science.

[14] Frank M, Hennenberg EM, Eyking A, Rünzi M, Gerken G, Scott P (2015). TLR signaling modulates side effects of anticancer therapy in the small intestine. J Immunol.

[15] Manichanh C, Varela E, Martinez C, Antolin M, Llopis M, Doré J (2008). The gut microbiota predispose to the pathophysiology of acute postradiotherapy diarrhea. Am J Gastroenterol.

[16] Nam YD, Kim HJ, Seo JG, Kang SW, Bae JW (2013). Impact of pelvic radiotherapy on gut microbiota of gynecological cancer patients revealed by massive pyrosequencing. PLoS ONE.

[17] Wang A, Ling Z, Yang Z, Kiela PR, Wang T, Wang C (2015). Gut microbial dysbiosis may predict diarrhea and fatigue in patients undergoing pelvic cancer radiotherapy: a pilot study. PLoS ONE.

[18] Mitra A, Grossman Biegert GW, Delgado AY, Karpinets TV, Solley TN, Mezzari MP (2020). Microbial diversity and composition is associated with patient-reported toxicity during chemoradiation therapy for cervical cancer. Int J Radiat Oncol Biol Phys.

[19] Saeed A, Eshrat FF, Umar S, Saeed A (2019). The duplex interaction of microbiome with chemoradiation and immunotherapy: potential implications for colorectal cancer. Curr Colorectal Cancer Rep.

[20] Alexander JL, Wilson ID, Teare J, Marchesi JR, Nicholson JK, Kinross JM (2017). Gut microbiota modulation of chemotherapy efficacy and toxicity. Nat Rev Gastroenterol Hepatol.

[21] Roy S, Trinchieri G (2017). Microbiota: a key orchestrator of cancer therapy. Nat Rev Cancer.

[22] Baskar R, Dai J, Wenlong N, Yeo R, Yeoh KW (2014). Biological response of cancer cells to radiation treatment. Front Mol Biosci.

[23] Kareva I (2019). Metabolism and gut microbiota in cancer immunoediting, CD8/Treg Ratios, immune cell homeostasis, and cancer (immuno)therapy: concise review. Stem Cells.

[24] Helmink BA, Khan MAW, Hermann A, Gopalakrishnan V, Wargo JA (2019). The microbiome, cancer, and cancer therapy. Nat Med.

[25] Chan SL (2020). Microbiome and cancer treatment: are we ready to apply in clinics?. Prog Mol Biol Transl Sci.

[26] Gately S (2019). Human microbiota and personalized cancer treatments: role of commensal microbes in treatment outcomes for cancer patients. Cancer Treat Res.

[27] Hekmatshoar Y, Rahbar Saadat Y, Hosseiniyan Khatibi SM, Ozkan T, Zununi Vahed F, Nariman-Saleh-Fam Z (2019). The impact of tumor and gut microbiotas on cancer therapy: beneficial or detrimental?. Life Sci.

[28] Wheeler KM, Liss MA (2019). The microbiome and prostate cancer risk. Curr Urol Rep.

[29] Scott AJ, Merrifield CA, Younes JA, Pekelharing EP (2018). Pre-, pro- and synbiotics in cancer prevention and treatment—a review of basic and clinical research. Ecancermedicalscience.

[30] Kim YS, Kim J, Park SJ (2015). High-throughput 16S rRNA gene sequencing reveals alterations of mouse intestinal microbiota after radiotherapy. Anaerobe.

[31] Jang BS, Chang JH, Chie EK, Kim K, Park JW, Kim MJ (2020). Gut Microbiome composition is associated with a pathologic response after preoperative chemoradiation in patients with rectal cancer. Int J Radiat Oncol Biol Phys.

[32] Touchefeu Y, Montassier E, Nieman K, Gastinne T, Potel G, Bruley des Varannes S (2014). Systematic review: the role of the gut microbiota in chemotherapy- or radiation-induced gastrointestinal mucositis: current evidence and potential clinical applications. Aliment Pharmacol Ther..

[33] Riquelme E, Zhang Y, Zhang L, Montiel M, Zoltan M, Dong W (2019). Tumor microbiome diversity and composition influence pancreatic cancer outcomes. Cell.

[34] Uribe-Herranz M, Rafail S, Beghi S, Gil-de-Gómez L, Verginadis I, Bittinger K (2020). Gut microbiota modulate dendritic cell antigen presentation and radiotherapy-induced antitumor immune response. J Clin Invest.

[35] Cui M, Xiao H, Li Y, Zhou L, Zhao S, Luo D (2017). Faecal microbiota transplantation protects against radiation-induced toxicity. EMBO Mol Med.

[36] Cui M, Xiao H, Luo D, Zhang X, Zhao S, Zheng Q (2016). Circadian rhythm shapes the gut microbiota affecting host radiosensitivity. Int J Mol Sci.

[37] Chan S, Rowbottom L, McDonald R, Bjarnason GA, Tsao M, Danjoux C (2017). Does the time of radiotherapy affect treatment outcomes? A review of the literature. Clin Oncol (R Coll Radiol).

[38] Kuwahara Y, Oikawa T, Ochiai Y, Roudkenar MH, Fukumoto M, Shimura T (2011). Enhancement of autophagy is a potential modality for tumors refractory to radiotherapy. Cell Death Dis.

[39] Digomann D, Kurth I, Tyutyunnykova A, Chen O, Löck S, Gorodetska I (2019). The CD98 heavy chain is a marker and regulator of head and neck squamous cell carcinoma radiosensitivity. Clin Cancer Res.

[40] Digomann D, Linge A, Dubrovska A (2019). SLC3A2/CD98hc, autophagy and tumor radioresistance: a link confirmed. Autophagy.

[41] Yu T, Guo F, Yu Y, Sun T, Ma D, Han J (2017). Fusobacterium nucleatum promotes chemoresistance to colorectal cancer by modulating autophagy. Cell.

[42] Kalluri R, Zeisberg M (2006). Fibroblasts in cancer. Nat Rev Cancer.

[43] Crawford PA, Gordon JI (2005). Microbial regulation of intestinal radiosensitivity. Proc Natl Acad Sci USA.

[44] Grootaert C, Van de Wiele T, Van Roosbroeck I, Possemiers S, Vercoutter-Edouart AS, Verstraete W (2011). Bacterial monocultures, propionate, butyrate and H_2_O_2_ modulate the expression, secretion and structure of the fasting-induced adipose factor in gut epithelial cell lines. Environ Microbiol.

[45] Korecka A, de Wouters T, Cultrone A, Lapaque N, Pettersson S, Doré J (2013). ANGPTL4 expression induced by butyrate and rosiglitazone in human intestinal epithelial cells utilizes independent pathways. Am J Physiol Gastrointest Liver Physiol.

[46] Demers M, Dagnault A, Desjardins J (2014). A randomized double-blind controlled trial: impact of probiotics on diarrhea in patients treated with pelvic radiation. Clin Nutr.

[47] Sonis ST, Elting LS, Keefe D, Peterson DE, Schubert M, Hauer-Jensen M (2004). Perspectives on cancer therapy-induced mucosal injury: pathogenesis, measurement, epidemiology, and consequences for patients. Cancer.

[48] Lalla RV, Sonis ST, Peterson DE (2008). Management of oral mucositis in patients who have cancer. Dent Clin North Am..

[49] Al-Qadami G, Van Sebille Y, Le H, Bowen J (2019). Gut microbiota: implications for radiotherapy response and radiotherapy-induced mucositis. Expert Rev Gastroenterol Hepatol.

[50] Ferreira MR, Muls A, Dearnaley DP, Andreyev HJ (2014). Microbiota and radiation-induced bowel toxicity: lessons from inflammatory bowel disease for the radiation oncologist. Lancet Oncol.

[51] Gerassy-Vainberg S, Blatt A, Danin-Poleg Y, Gershovich K, Sabo E, Nevelsky A (2018). Radiation induces proinflammatory dysbiosis: transmission of inflammatory susceptibility by host cytokine induction. Gut.

[52] Goudarzi M, Mak TD, Jacobs JP, Moon BH, Strawn SJ, Braun J (2016). An integrated multi-omic approach to assess radiation injury on the host-microbiome axis. Radiat Res.

[53] Montassier E, Batard E, Massart S, Gastinne T, Carton T, Caillon J (2014). 16S rRNA gene pyrosequencing reveals shift in patient faecal microbiota during high-dose chemotherapy as conditioning regimen for bone marrow transplantation. Microb Ecol.

[54] Neemann K, Freifeld A (2017). Clostridium difficile-associated diarrhea in the oncology patient. J Oncol Pract.

[55] van Vliet MJ, Harmsen HJ, de Bont ES, Tissing WJ (2010). The role of intestinal microbiota in the development and severity of chemotherapy-induced mucositis. PLoS Pathog.

[56] Wardill HR, Gibson RJ, Van Sebille YZA, Secombe KR, Coller JK, White IA (2016). Irinotecan-induced gastrointestinal dysfunction and pain are mediated by common TLR4-dependent mechanisms. Mol Cancer Ther.

[57] Riehl T, Cohn S, Tessner T, Schloemann S, Stenson WF (2000). Lipopolysaccharide is radioprotective in the mouse intestine through a prostaglandin-mediated mechanism. Gastroenterology.

[58] Riehl TE, Newberry RD, Lorenz RG, Stenson WF (2004). TNFR1 mediates the radioprotective effects of lipopolysaccharide in the mouse intestine. Am J Physiol Gastrointest Liver Physiol.

[59] Egan LJ, Eckmann L, Greten FR, Chae S, Li ZW, Myhre GM (2004). IκB-kinaseβ-dependent NF-κB activation provides radioprotection to the intestinal epithelium. Proc Natl Acad Sci USA.

[60] Gori S, Inno A, Belluomini L, Bocus P, Bisoffi Z, Russo A, Arcaro G (2019). Gut microbiota and cancer: how gut microbiota modulates activity, efficacy and toxicity of antitumoral therapy. Crit Rev Oncol Hematol.

[61] Pandey KR, Naik SR, Vakil BV (2015). Probiotics, prebiotics and synbiotics—a review. J Food Sci Technol.

[62] Zhang M, Sun K, Wu Y, Yang Y, Tso P, Wu Z (2017). Interactions between intestinal microbiota and host immune response in inflammatory bowel disease. Front Immunol.

[63] Cousin FJ, Jouan-Lanhouet S, Théret N, Brenner C, Jouan E, Le Moigne-Muller G (2016). The probiotic Propionibacterium freudenreichii as a new adjuvant for TRAIL-based therapy in colorectal cancer. Oncotarget.

[64] Lenoir M, Del Carmen S, Cortes-Perez NG, Lozano-Ojalvo D, Muñoz-Provencio D, Chain F (2016). Lactobacillus casei BL23 regulates Treg and Th17 T-cell populations and reduces DMH-associated colorectal cancer. J Gastroenterol.

[65] Del Carmen S, de Moreno de LeBlanc A, Levit R, Levit R, Azevedo V, Langella P (2017). Anti-cancer effect of lactic acid bacteria expressing antioxidant enzymes or IL-10 in a colorectal cancer mouse model. Int Immunopharmacol..

[66] Mohania D, Kansal VK, Kruzliak P, Kumari A (2014). Probiotic Dahi containing *Lactobacillus acidophilus* and *Bifidobacterium bifidum* modulates the formation of aberrant crypt foci, mucin-depleted foci, and cell proliferation on 1,2-dimethylhydrazine-induced colorectal carcinogenesis in Wistar rats. Rejuvenation Res.

[67] Ho CL, Tan HQ, Chua KJ, Kang A, Lim KH, Ling KL (2018). Engineered commensal microbes for diet-mediated colorectal-cancer chemoprevention. Nat Biomed Eng.

[68] Franko J, Raman S, Krishnan N, Frankova D, Tee MC, Brahmbhatt R (2019). Randomized trial of perioperative probiotics among patients undergoing major abdominal operation. J Am Coll Surg.

[69] Chowdhury AH, Adiamah A, Kushairi A, Varadhan KK, Krznaric Z, Kulkarni AD (2020). Perioperative probiotics or synbiotics in adults undergoing elective abdominal surgery: a systematic review and meta-analysis of randomized controlled trials. Ann Surg.

[70] Casas-Solís J, Huizar-López MDR, Irecta-Nájera CA, Pita-López ML, Santerre A (2019). Immunomodulatory effect of lactobacillus casei in a murine model of colon carcinogenesis. Probiotics Antimicrob Proteins.

[71] Krebs B (2016). Prebiotic and synbiotic treatment before colorectal surgery–randomised double blind trial. Coll Antropol.

[72] Krebs B, Horvat M, Golle A, Krznaric Z, Papeš D, Augustin G (2013). A randomized clinical trial of synbiotic treatment before colorectal cancer surgery. Am Surg.

[73] Worthley DL, Le Leu RK, Whitehall VL, Conlon M, Christophersen C, Belobrajdic D (2009). A human, double-blind, placebo-controlled, crossover trial of prebiotic, probiotic, and synbiotic supplementation: effects on luminal, inflammatory, epigenetic, and epithelial biomarkers of colorectal cancer. Am J Clin Nutr.

[74] Lamichhane P, Maiolini M, Alnafoosi O, Hussein S, Alnafoosi H, Umbela S (2020). Colorectal cancer and probiotics: are bugs really drugs?. Cancers (Basel).

[75] Liu MM, Li ST, Shu Y, Zhan HQ (2017). Probiotics for prevention of radiation-induced diarrhea: a meta-analysis of randomized controlled trials. PLoS ONE.

[76] Gibson GR, Hutkins R, Sanders ME, Prescott SL, Reimer RA, Salminen SJ (2017). Expert consensus document: the international scientific association for probiotics and prebiotics (ISAPP) consensus statement on the definition and scope of prebiotics. Nat Rev Gastroenterol Hepatol.

[77] Qamar TR, Syed F, Nasir M, Rehman H, Zahid MN, Liu RH (2016). Novel combination of prebiotics galacto-oligosaccharides and inulin-inhibited aberrant crypt foci formation and biomarkers of colon cancer in Wistar rats. Nutrients.

[78] Skiba MB, Kohler LN, Crane TE, Jacobs ET, Shadyab AH, Kato I (2019). The association between prebiotic fiber supplement use and colorectal cancer risk and mortality in the women's health initiative. Cancer Epidemiol Biomark Prev.

[79] Kaźmierczak-Siedlecka K, Daca A, Fic M, van de Wetering T, Folwarski M, Makarewicz W (2020). Therapeutic methods of gut microbiota modification in colorectal cancer management—fecal microbiota transplantation, prebiotics, probiotics, and synbiotics. Gut Microbes.

[80] Eid N, Osmanova H, Natchez C, Walton G, Costabile A, Gibson G (2015). Impact of palm date consumption on microbiota growth and large intestinal health: a randomised, controlled, cross-over, human intervention study. Br J Nutr.

[81] Le Leu RK, Winter JM, Christophersen CT, Young GP, Humphreys KJ, Hu Y (2015). Butyrylated starch intake can prevent red meat-induced O6-methyl-2-deoxyguanosine adducts in human rectal tissue: a randomised clinical trial. Br J Nutr.

[82] Molan AL, Liu Z, Plimmer G (2014). Evaluation of the effect of blackcurrant products on gut microbiota and on markers of risk for colon cancer in humans. Phytother Res.

[83] Windey K, De Preter V, Huys G, Broekaert WF, Delcour JA, Louat T (2015). Wheat bran extract alters colonic fermentation and microbial composition, but does not affect faecal water toxicity: a randomised controlled trial in healthy subjects. Br J Nutr.

[84] Mehta RS, Nishihara R, Cao Y, Song M, Mima K, Qian ZR (2017). Association of dietary patterns with risk of colorectal cancer subtypes classified by Fusobacterium nucleatum in tumor tissue [published correction appears in JAMA Oncol. 2019;5(4):579]. JAMA Oncol..

[85] Limburg PJ, Mahoney MR, Ziegler KL, Sontag SJ, Schoen RE, Benya R (2011). Randomized phase II trial of sulindac, atorvastatin, and prebiotic dietary fiber for colorectal cancer chemoprevention. Cancer Prev Res (Phila).

[86] Martin OC, Lin C, Naud N, Tache S, Raymond-Letron I, Corpet DE (2015). Antibiotic suppression of intestinal microbiota reduces heme-induced lipoperoxidation associated with colon carcinogenesis in rats. Nutr Cancer.

[87] Ushijima H, Horyozaki A, Maeda M (2016). Anisomycin-induced GATA-6 degradation accompanying a decrease of proliferation of colorectal cancer cell. Biochem Biophys Res Commun.

[88] Prabhu VV, Hong B, Allen JE, Zhang S, Lulla AR, Dicker DT (2016). Small-molecule prodigiosin restores p53 tumor suppressor activity in chemoresistant colorectal cancer stem cells via c-Jun-mediated ΔNp73 inhibition and p73 activation. Cancer Res.

[89] Klose J, Eissele J, Volz C, Schmitt S, Ritter A, Ying S (2016). Salinomycin inhibits metastatic colorectal cancer growth and interferes with Wnt/β-catenin signaling in CD133+ human colorectal cancer cells. BMC Cancer.

[90] Bullman S, Pedamallu CS, Sicinska E, Clancy TE, Zhang X, Cai D (2017). Analysis of Fusobacterium persistence and antibiotic response in colorectal cancer. Science.

[91] Montrose DC, Zhou XK, McNally EM, Sue E, Yantiss RK, Gross SS (2016). Celecoxib alters the intestinal microbiota and metabolome in association with reducing polyp burden. Cancer Prev Res (Phila).

[92] Yu YN, Yu TC, Zhao HJ, Sun TT, Chen HM, Chen HY (2015). Berberine may rescue *Fusobacterium nucleatum*-induced colorectal tumorigenesis by modulating the tumor microenvironment. Oncotarget.

[93] Wu M, Wu Y, Deng B, Li J, Cao H, Qu Y (2016). Isoliquiritigenin decreases the incidence of colitis-associated colorectal cancer by modulating the intestinal microbiota. Oncotarget.

[94] McFadden RM, Larmonier CB, Shehab KW, Midura-Kiela M, Ramalingam R, Harrison CA (2015). The role of curcumin in modulating colonic microbiota during colitis and colon cancer prevention. Inflamm Bowel Dis.

[95] Tomasello G, Mazzola M, Jurjus A, Cappello F, Carini F, Damiani P (2017). The fingerprint of the human gastrointestinal tract microbiota: a hypothesis of molecular mapping. J Biol Regul Homeost Agents.

[96] El Rakaiby M, Dutilh BE, Rizkallah MR, Boleij A, Cole JN, Aziz RK (2014). Pharmacomicrobiomics: the impact of human microbiome variations on systems pharmacology and personalized therapeutics. OMICS.

[97] Cao H, Xu M, Dong W, Deng B, Wang S, Zhang Y (2017). Secondary bile acid-induced dysbiosis promotes intestinal carcinogenesis. Int J Cancer.

[98] Castellarin M, Warren RL, Freeman JD, Dreolini L, Krzywinski M, Strauss J (2012). Fusobacterium nucleatum infection is prevalent in human colorectal carcinoma. Genome Res.

[99] Ding X, Li Q, Li P, Chen X, Xiang L, Bi L (2020). Fecal microbiota transplantation: A promising treatment for radiation enteritis?. Radiother Oncol.

[100] Xiao HW, Cui M, Li Y, Dong JL, Zhang SQ, Zhu CC (2020). Gut microbiota-derived indole 3-propionic acid protects against radiation toxicity via retaining acyl-CoA-binding protein. Microbiome.

[101] Pamer EG (2014). Fecal microbiota transplantation: effectiveness, complexities, and lingering concerns. Mucosal Immunol.

[102] Borody TJ, Paramsothy S, Agrawal G (2013). Fecal microbiota transplantation: indications, methods, evidence, and future directions. Curr Gastroenterol Rep.

[103] Hoffmann D, Palumbo F, Ravel J, Roghmann MC, Rowthorn V, von Rosenvinge E (2017). Improving regulation of microbiota transplants. Science.

[104] Ronda C, Chen SP, Cabral V, Yaung SJ, Wang HH (2019). Metagenomic engineering of the mammalian gut microbiome in situ. Nat Methods.

[105] Song W, Anselmo AC, Huang L (2019). Nanotechnology intervention of the microbiome for cancer therapy. Nat Nanotechnol.

[106] Chowdhury S, Castro S, Coker C, Hinchliffe TE, Arpaia N, Danino T (2019). Programmable bacteria induce durable tumor regression and systemic antitumor immunity. Nat Med.

[107] Sola-Oladokun B, Culligan EP, Sleator RD (2017). Engineered probiotics: applications and biological containment. Annu Rev Food Sci Technol.

[108] Kommineni S, Bretl DJ, Lam V, Chakraborty R, Hayward M, Simpson P (2015). Bacteriocin production augments niche competition by enterococci in the mammalian gastrointestinal tract. Nature.

[109] Özel B, Şimşek Ö, Akçelik M, Saris PEJ (2018). Innovative approaches to nisin production. Appl Microbiol Biotechnol.

[110] Suwan K, Yata T, Waramit S, Przystal JM, Stoneham CA, Bentayebi K (2019). Next-generation of targeted AAVP vectors for systemic transgene delivery against cancer. Proc Natl Acad Sci USA.

[111] Kingwell K (2015). Bacteriophage therapies re-enter clinical trials. Nat Rev Drug Discov.

[112] Costea PI, Coelho LP, Sunagawa S, Munch R, Huerta-Cepas J, Forslund K (2017). Subspecies in the global human gut microbiome. Mol Syst Biol.

[113] Wirbel J, Pyl PT, Kartal E, Zych K, Kashani A, Milanese A (2019). Meta-analysis of fecal metagenomes reveals global microbial signatures that are specific for colorectal cancer. Nat Med.

[114] Zhang X, Figeys D (2019). Perspective and guidelines for metaproteomics in microbiome studies. J Proteome Res.

[115] Lloyd-Price J, Arze C, Ananthakrishnan AN, Schirmer M, Avila-Pacheco J, Poon TW (2019). Multi-omics of the gut microbial ecosystem in inflammatory bowel diseases. Nature.

[116] Yimagou EK, Baudoin JP, Abdallah RA, Di Pinto F, Bou Khalil JY, Raoult D (2020). Full-repertoire comparison of the microscopic objects composing the human gut microbiome with sequenced and cultured communities. J Microbiol.

[117] Haase S, Haghikia A, Wilck N, Müller DN, Linker RA (2018). Impacts of microbiome metabolites on immune regulation and autoimmunity. Immunology.

[118] Morrison DJ, Preston T (2016). Formation of short chain fatty acids by the gut microbiota and their impact on human metabolism. Gut Microbes.

